# Efficacy and safety of a popular thermogenic drink after 28 days of ingestion

**DOI:** 10.1186/1550-2783-5-19

**Published:** 2008-10-24

**Authors:** Michael D Roberts, Vincent J Dalbo, Scott E Hassell, Jeffrey R Stout, Chad M Kerksick

**Affiliations:** 1 Department of Health and Exercise Science, University of Oklahoma, 1401 Asp Ave, Norman, OK, USA

## Abstract

**Background:**

We have recently demonstrated that consuming a thermogenic drink (TD) acutely increases energy expenditure and serum markers of lipolysis in healthy, college-aged individuals. The purpose of this study was to determine if consuming TD over 28 days affects its acute thermogenic and lipolytic effects as well as body composition and clinical chemistry safety markers.

**Methods:**

Sixty healthy, males (mean ± SE; 23 ± 1 years, 177 ± 2 cm, 81.7 ± 2.1 kg, 22.8 ± 1.4% body fat; n = 30) and females (23 ± 1 years, 166 ± 2 cm, 62.1 ± 1.8 kg, 28.3 ± 1.4% body fat; n = 30) reported to the laboratory on day 0 (T1) for determination of body composition, resting energy expenditure (REE) as well as glycerol and free fatty acid (FFA) levels before and after ingesting either 336 ml of TD or a non-caloric, non-caffeinated placebo (PLA) drink. Following day 0, participants supplemented daily with 336 ml·day^-1 ^of either TD or PLA and repeated identical testing procedures on day 28 (T2). Day 28 area under the curve (AUC) values were calculated for REE, FFA, and glycerol. Day 28 acute data and prolonged AUC comparisons between groups were analyzed using ANOVAs with repeated measures.

**Results:**

Percent body fat (p = 0.02) and fat mass (p = 0.01) decreased in the TD group compared to the PLA group after 28 days. Day 28 FFA AUC values (p = 0.048) were greater in the TD group compared to the PLA group. There was no significant difference in day 28 REE AUC values (p = 0.30) or glycerol AUC values (p = 0.21), although a significant increase in REE values in the PLA group may have confounded these findings. There were no differences between groups concerning blood and clinical safety markers.

**Conclusion:**

Within-group elevations in FFA and REE values in the TD group were still evident following a 28-day supplementation period which may contribute to the observed decrements in %BF. Further, prolonged TD supplementation did not alter the assessed clinical safety markers. Future studies should examine the synergistic and independent effects of the active ingredients in addition to effects of longer ingestion periods of TD ingestion with or without exercise at promoting and sustaining changes in body composition.

## Background

The rising prevalence of obesity has incited widespread interest in over-the-counter (OTC) thermogenic weight loss products. Common OTC weight loss supplements are made up of proprietary blends containing caffeine, ephedrine alkaloids, epigallocatechin (EGCG), and other agents such as fiber [1,2]. Nutritional supplements containing one or more of these aforementioned ingredients purportedly facilitate thermogenesis and lipolysis. For instance, administering as little as 100 mg of caffeine has been shown to acutely increase energy expenditure by 3–4% in humans [3]. Caffeine ingestion has also been shown to increase circulating markers of lipolysis including glycerol and free fatty acids (FFAs) [4,5]. Additionally, administering catechins to humans has been shown to acutely increase energy expenditure by approximately 365 kcal over a 4-hour period [6]. Our lab has previously demonstrated a popular OTC thermogenic beverage (TD) containing caffeine and EGCG to be effective in acutely increasing resting energy expenditure (REE) and FFA levels up to 180 minutes following consumption [7]. However, it is currently unknown if ingesting TD over longer durations of daily use reduces its thermogenic and lipolytic effects.

Evidence exists demonstrating that the catecholamine response to caffeine following its prolonged use is blunted in humans [8] leading to speculation that any physiological impact will be negated as daily use continues. Other *in vitro *evidence suggests that prolonged caffeine exposure up-regulates the expression of lipolysis-inhibiting adenosine (A_1_) receptors in adipocytes [9]. However, applied research has demonstrated that a more prolonged use (i.e., 8 weeks) of a caffeine/EGCG supplement is effective in reducing body mass [6] suggesting that these ingredients sustain their thermogenic effects during prolonged supplementation periods. Similarly, Belza et al. [6] have also recently demonstrated that increases in resting metabolism were sustained after 8 weeks of supplementing participants with a mixture of tyrosine, capsaicin, catechins, and caffeine. Although this evidence suggests that caffeine/EGCG supplements sustains a thermogenic effect after prolonged use, it is currently unknown whether ingesting TD over prolonged periods blunts the thermogenic and lipolytic properties that have been previously reported. Thus, the purpose of this investigation was to determine if prolonged TD use affected the drink's thermogenic and lipolytic actions. A secondary objective was to examine the supplement's safety by measuring clinical safety markers and its efficacy in reducing fat mass and/or percent body fat following 28 days of supplementation.

## Methods

### Participants

Sixty healthy, college-aged males (mean ± SE; 23 ± 1 years, 177 ± 2 cm, 81.7 ± 2.1 kg, 22.8 ± 1.4% body fat; n = 30) and females (23 ± 1 years, 166 ± 2 cm, 62.1 ± 1.8 kg, 28.3 ± 1.4% body fat; n = 30) were informed of the experimental procedures and signed informed consent statements and medical history forms in adherence with the Institutional Review Board of the University of Oklahoma and the American College of Sports Medicine (ACSM) prior to data collection. Participants were excluded if they: 1) had any history of metabolic, hypertension, hepatorenal, musculoskeletal, autoimmune, or neurological disease; 2) were currently taking thyroid, antihyperlipidemic, hypoglycemic, anti-hypertensive, or androgenic medications; and 3) had taken nutritional supplements that may affect metabolism [i.e., over 100 mg·day^-1 ^of caffeine, ephedrine alkaloids, guggulsterones, etc.] and/or muscle mass [i.e. creatine, protein/amino acids, androstenedione, dihydroepiandrosterone, etc.] within three months of the starting the study.

### Study design

Eligible participants were familiarized to the study protocol via a verbal and written explanation of the study design. Participants were instructed to refrain from strenuous exercise for 24 hours, and fast for 12 hours prior to baseline (i.e., day 0) and follow-up (i.e., following 28 days of ingestion) testing. This study employed a single-blind, placebo-controlled, parallel study design whereby participants were grouped into clusters by gender and fat free mass for assignment into one of two groups prior to day 0 testing.

### Procedures

During days 0 and 28 each participant reported to the laboratory after a 12-hour fast. Height was measured using standard anthropometry. Body composition parameters including body mass, lean mass, fat mass, and body fat percentage (%BF) were determined using air plethysmography (Life Measurement Inc. Concord, CA) (BOD POD). In short, participants performed the test wearing a tight-fitting bathing suit and a swim cap whereby he/she sat motionless in the device for 20 seconds to assess body volume and subsequently breathed into an internal breathing tube to correct for thoracic gas volume. Integrated software adjusted body volume for thoracic gas volume and calculated body percentage using the Brozek equation [10]. Our laboratory has previously demonstrated a total error of measurement of 0.66 %BF using the BOD POD device. Using a sub-sample of participants from this investigation yielded a test-retest intra-class coefficient of > 0.99 (p < 0.001) for body fat percentage determination using the BOD POD. Blood pressure and resting heart rate were then determined using an electronic sphygmomanometer (HEM-757, Omron HealthCare Inc., Vernon Hills, IL). Participants then had his/her REE determined using indirect calorimetry (True One 2400 Metabolic Measurement System, ParvoMedics Inc., Sandy, UT) whereby he/she rested in a supine position under a clear, plastic metabolic hood with a plastic drape over their shoulders and torso for 15 minutes. REE values were recorded every 15 seconds and averaged over the 10–15 minute time period. Using a sub-sample of participants from this investigation, the mean intra-class coefficient of collected VO_2 _in L·min^-1 ^was 0.942 (p < 0.001).

Participants then donated approximately 20 ml of fasting blood using standard venipuncture techniques. Samples were subsequently assayed for hematological, clinical chemistry panels, glycerol, and FFAs. Two 6 ml serum separation tubes and one 5 ml K_3_EDTA vacutainer tube were inserted into the vacutainer holder for blood collection in succession using multiple sample phlebotomy techniques. The serum separation tubes were centrifuged at room temperature for 15 minutes at 3,500 rev·minute^-1^, the serum supernatant was transferred into two microcentrifuge tubes, and the serum samples were stored at -20°C for subsequent metabolite analyses. Serum and the whole blood from the K_3 _EDTA tube were used to analyze serum clinical chemistry markers and complete blood counts (CBC), respectively, using automated analyzers from a commercial diagnostic laboratory. Following the baseline REE determination and blood donation, each participant randomly ingested 336 ml of either TD or a non-caloric, non-caffeinated PLA. Participants then underwent REE determinations at 60, 120, and 180 minutes following drink ingestion and donated 6 ml of blood for glycerol and FFA determination using standard venipuncture techniques at 30, 60, 120, and 180 minutes following drink ingestion.

Following day 0 testing, participants completed a 27-day supplementation period as described below. Participants then returned on day 28 and repeated day 0 testing procedures above whereby he/she reported to the laboratory during the same time as his/her previous session.

### Supplementation protocol and dietary monitoring

In a single-blind fashion, participants were assigned to ingest one 336 ml Celsius (Celsius, Inc., Delray Beach, FL) [10 kcal, Thermogenic Proprietary Blend: caffeine (200 mg), guarana extract (seed), green tea leaf extract (leaf) standardized to 10% EGCG (250 mg), glucoronolactone, ginger extract (root), taurine] or a non-caloric/non-caffeinated PLA per day following baseline testing. Supplements were packaged in generic bottles by Celsius, Inc. Compliance was monitored by having participants pick up supplements from the laboratory on a weekly basis as well as completing a supplement log throughout the duration of the study. Prior to the study and during the weekly visits, participants were provided a list of common drinks and foods that contained high levels of caffeine and were instructed to avoid regular consumption of these foods. Additionally, participants were instructed to maintain the same physical activity levels that they had prior to the study. Dietary intake was monitored with 2-day dietary recalls at the beginning and end of the supplementation period to ensure between-group caloric and caffeine homogeneity. Caloric and caffeine intake was assessed using the Food Processor III Nutrition Software version 6.8 (ESHA Nutrition Research, Salem, OR).

### Serum and whole blood analyses

Serum and whole blood samples were used to evaluate clinical safety during the supplementation protocol. Serum samples were assayed at a commercial diagnostic laboratory using automated clinical chemistry and hematology analyzers for comprehensive metabolic panels including glucose, triglycerides, cholesterol, HDL cholesterol, LDL cholesterol, total protein, blood urea nitrogen (BUN), creatinine, BUN/creatinine ratio, albumin, globulin, sodium, potassium, chloride, calcium, carbon dioxide, total bilirubin, aspartate aminotransferase (AST), alanine aminotransferase (ALT), gamma-glutamyl transpeptidase (GGT), and alkaline phosphatase (ALP). Whole blood was analyzed for red cell counts, hemoglobin, hematocrit, mean cell volume, mean corpuscular hemoglobin, mean corpuscular hemoglobin concentration, red cell distribution width, white blood cell counts, neutrophils, lymphocytes, monocytes, eosinophils, and basophils.

Stored serum samples were later assayed for glycerol and FFAs. An enzymatic oxygen-rate analyzer (Analox GM7, Analox Instruments USA Inc., Lunenburg MA) was used to analyze serum glycerol concentrations. A commercial spectrophotometric assay was used to analyze serum FFAs (Roche Diagnostics Corporation, Indianapolis, IN). All samples were assayed in duplicate. Assay precision [i.e., coefficient of variation (C_*V*_)] and accuracy (i.e., percent of unrecovery which is the percentage deviation from 100%) were calculated using glycerol and FFA control samples by 8 replicate determinations per analyte. In the current investigation, the C_*V *_and percent of unrecovery using a 240 μM glycerol and 0.35 mM free-fatty acid control serum was 2.5% and 1.2%, as well as 8.9% and 3.6%, respectively, which is in accordance with previously accepted guidelines [11,12].

### Statistical analysis

Area under the curve (AUC) analyses using the trapezoidal method was performed for REE, glycerol and FFA on days 0 and 28. Between-group differences were compared using independent t-tests, while between-group changes over time were compared using separate 2 × 2 (group × testing session) ANOVAs with repeated measures. Changes in body mass, fat-free mass, fat mass, %BF, caffeine, and caloric intake were analyzed also using separate 2 × 2 (group × testing session) ANOVA with repeated measures. Due to the fact that caffeine is the primary active ingredient of TD, correlations were performed between caffeine dosage per kg bodyweight during day 28 versus metabolic responses (i.e., integrated AUCs for REE, glycerol, and FFA). Furthermore, correlations were performed examining day 0 caffeine dosage and changes in body fat within the TD group. Serum clinical chemistry markers and CBCs were analyzed using separate 2 × 2 (group × session) ANOVA with repeated measures. All statistical analyses were performed using SPSS (version 14.0, SPSS Inc., Chicago, IL). In circumstances where sphericity within groups could not be assumed due to large within-group variances, the Hunyhs-Feldt epsilon correction factor was used to adjust within group F-ratios. For all significant group × time interactions, additional pair-wise comparisons were used to assess which time points yielded statistical significance between groups. Significance for all statistical analyses was determined using an alpha level of 0.05.

## Results

### Side effects

Sixty participants from an original pool of sixty-two completed the study. One participant in the TD group withdrew from the study due to reoccurring symptoms of gastrointestinal distress whereas one participant in the PLA group withdrew from the study following baseline testing due to not wanting to ingest the artificial sweetener within the PLA drink over 28 days.

### Nutritional intake

All dietary intake was normalized to body mass and statistical comparisons revealed that the PLA group significantly increased their carbohydrate intake following the 28-day intervention (p = 0.02; Table 1). There was no difference in baseline caffeine intake prior to the study with the TD group reporting an average intake of 0.2 ± 0.1 mg·kg^-1^·day^-1 ^and the PLA group reporting an intake of 0.2 ± 0.1 mg·kg^-1^·day^-1 ^(p = 0.63). The daily dosage of caffeine administered to the TD group on day 0 was not standardized to body mass and in *post-hoc *fashion was estimated to be 2.5 ± 0.1 mg·kg^-1^·day^-1 ^for males and 3.2 ± 0.1 mg·kg^-1^·day^-1 ^for females (p < 0.0001).

**Table 1:** Body composition and dietary intake

Variable	Group	Day 0	Day 28	Within group	Between group*
Body mass (kg)	TD PLA	72.6 ± 2.6 71.2 ± 2.7	72.4 ± 2.6 71.6 ± 2.7	p = 0.20 p = 0.20	Group: 0.77 Time: 0.61 G × T: 0.08
Fat-free mass (kg)	TD PLA	53.7 ± 1.9 53.1 ± 2.2	53.8 ± 2.3 52.9 ± 2.3	p = 0.62 p = 0.65	Group: 0.79 Time: 0.99 G × T: 0.50
Fat mass (kg)	TD PLA	18.9 ± 1.5 18.1 ± 1.3	18.3 ± 1.5^§^ 18.4 ± 1.2	p < 0.05 p = 0.20	Group: 0.87 Time: 0.57 G × T: < 0.05
BF% (%)	TD PLA	25.6 ± 1.4 25.1 ± 1.5	25.4 ± 1.5 25.9 ± 1.5	p = 0.09 p = 0.13	Group: 0.89 Time: 0.99 G × T: < 0.05
Caffeine intake (mg·kg^-1^·d^-1^)^†^	TD PLA	0.2 ± 0.1 0.2 ± 0.1	2.9 ± 0.2^§‡^ 0.2 ± 0.1	p < 0.001 p = 0.39	Group: < 0.001 Time: < 0.001 G × T: < 0.001
Caloric intake (kcal·kg^-1^·d^-1^)	TD PLA	26 ± 8 28 ± 13	24 ± 10 26 ± 8	p = 0.22 p = 0.17	Group: 0.58 Time: 0.08 G × T: 0.95
Protein intake (g·kg·d^-1^)	TD PLA	1.2 ± 0.4 1.1 ± 0.4	1.3 ± 0.8 1.1 ± 0.4	p = 0.27 p = 0.21	Group: 0.97 Time: 0.17 G × T: 0.97
Carbohydrate intake^†^ (g·kg·d^-1^)	TD PLA	3.3 ± 1.2 2.9 ± 1.3	3.7 ± 1.7 3.1 ± 1.2^§^	p = 0.07 p < 0.05	Group: 0.64 Time: < 0.01 G × T: 0.57
Fat intake (g·kg·d^-1^)	TD PLA	0.9 ± 0.4 1.1 ± 0.6	0.9 ± 0.4 1.0 ± 0.4	p = 0.57 p = 0.82	Group: 0.28 Time: 0.85 G × T: 0.69

TD, thermogenic drink; PLA, placebo

Values are expressed as means ± SE

BF%, body fat percentage

^†^Significant main effect for time, p < 0.05

^§^Different from Day 0, p < 0.05

^‡ ^Different from PLA, p < 0.001

*measured using a 2 × 2 (group × time) repeated measures ANOVA

### Body composition

Changes in body composition over the 28-day protocol are presented in Table 1. Repeated measures ANOVA revealed a significant group × time interaction in %BF between groups (p = 0.02). Within group analysis in the TD group revealed a trend %BF to decrease (p = 0.09), whereas there was no change in the PLA group (p = 0.20). Repeated measures ANOVA also revealed a significant group × time interaction in fat mass between groups (p = 0.01). Furthermore, there was a significant decrease in fat mass in the TD group from day 0 to 28 (p = 0.03), whereas there was no change in the PLA group (p = 0.20). Caffeine dosage within the TD group was weakly correlated with the day 0 to day 28 change in fat mass (*r *= 0.37, p = 0.04).

### Acute ingestion response after 28 days

**Figure 1:**
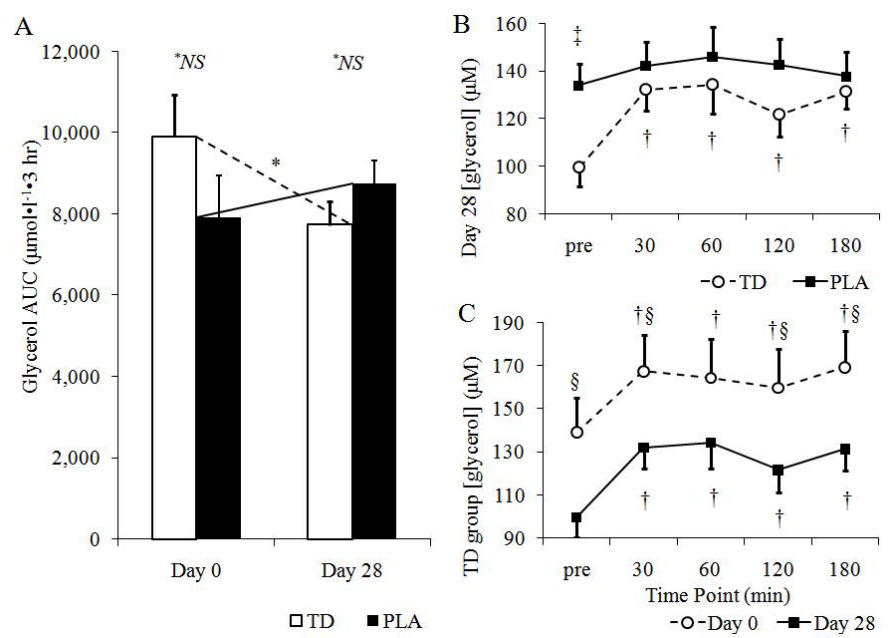
**Group glycerol AUC values (*A*), between group differences in [glycerol] on day 28 (*B*), and day 0 versus day 28 comparison of [glycerol] in the TD group; all data is expressed as means ± SE; TD, thermogenic drink group (n = 30); PLA, placebo (n = 30); AUC, incremental area under the curve**. Sub-figure A: *^NS ^no significant differences between groups, p > 0.05; *significant decrease within TD following 28 days of supplementation, p < 0.05. Sub-figure B: ^‡^TD < PLA, p < 0.05; ^†^pre-ingestion value < post-ingestion value, p < 0.05. Sub-figure C: ^§^significant difference within the TD group between days 0 and 28, p < 0.05; ^†^pre-ingestion value < post-ingestion value, p < 0.05

There were no differences in day 28 glycerol AUC values between groups (p = 0. 21; Figure 1). Additional independent t-tests at each time point revealed significantly higher values in the PLA group prior to ingestion (p = 0.007), whereas there were no differences between groups 30, 60, 120, and 180 minutes after ingestion (p > 0.05). Interestingly, there were significant within-group increases in glycerol at all time points compared to baseline within the TD group (p < 0.05), whereas there were no within-group changes in the PLA group. There was also a weak, but significant correlation between caffeine dosage (corrected for body mass) within the TD group on day 28 and glycerol AUC (*r *= 0.33, p = 0.01).

**Figure 2:**
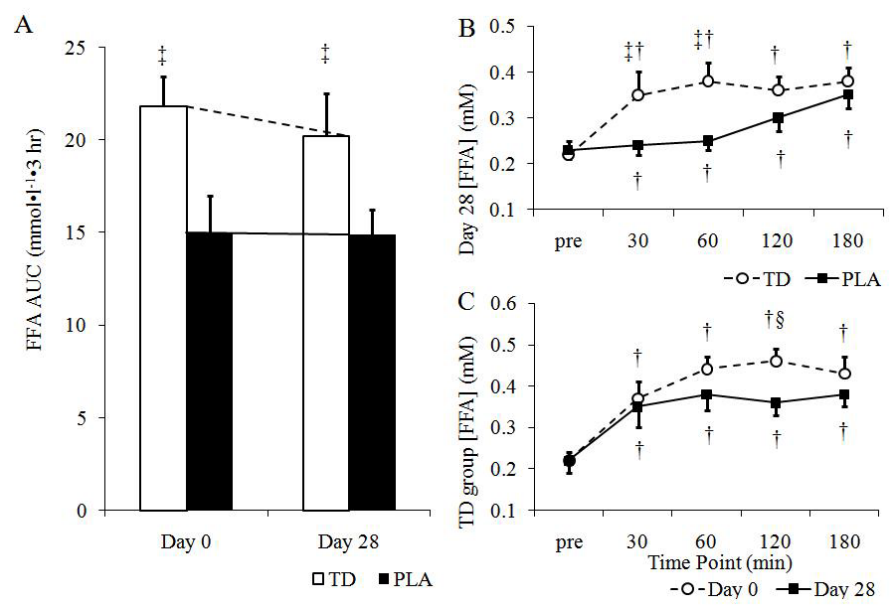
**Group FFA AUC values (*A*), between group differences in [FFA] on day 28 (*B*), and day 0 versus day 28 comparison of [FFA] in the TD group; all data is expressed as means ± SE; TD, thermogenic drink group (n = 30); PLA, placebo (n = 30); AUC, incremental area under the curve**. Sub-figure A: ^‡^TD > PLA, p < 0.05. Sub-figure B: ^‡^TD > PLA, p < 0.05; ^†^pre-ingestion value < post-ingestion value, p < 0.05. Sub-figure C: ^§^significant difference within the TD group between days 0 and 28, p < 0.05; ^†^pre-ingestion value < post-ingestion value, p < 0.05

**Figure 3:**
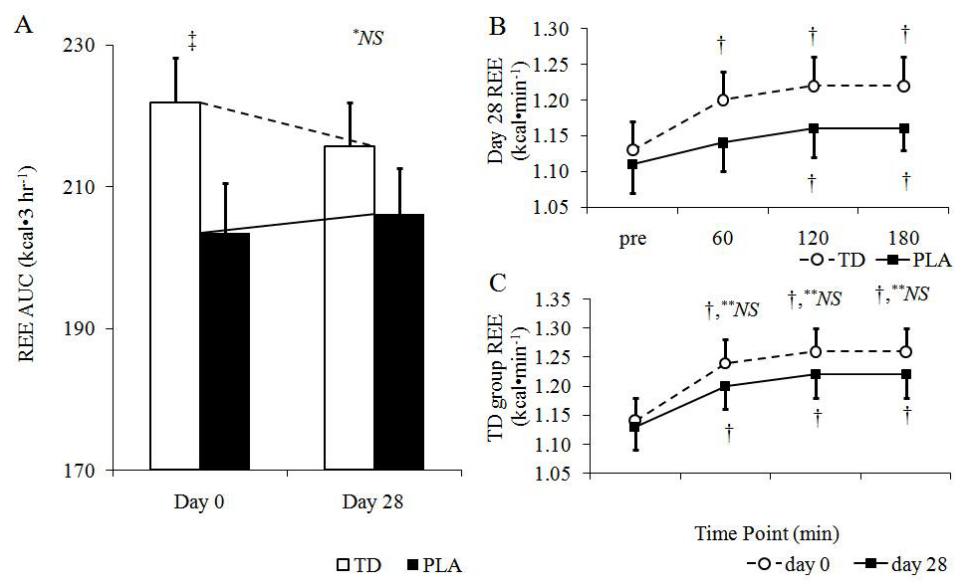
**Group REE AUC values (*A*), between group differences in REE on day 28 (*B*), and day 0 versus day 28 comparison of REE in the TD group; all data is expressed as means ± SE; TD, thermogenic drink group (n = 30); PLA, placebo (n = 30); AUC, incremental area under the curve**. Sub-figure A: *^NS ^no significant differences between groups, p > 0.05; ^‡^TD > PLA, p < 0.05; *significant decrease within groups TD following 28 days of supplementation, p < 0.05. Sub-figure B: ^†^pre-ingestion value < post-ingestion value for TD and PLA, p < 0.05. Sub-figure C: ^†^pre-ingestion value < post-ingestion value at Day 0 and Day 28 for TD only, p < 0.05; **^NS ^no significant differences between days 0 and 28, p > 0.05

Similarly, day 28 FFA AUC values were significantly greater in the TD group compared to the PLA group (p = 0.048; Figure 2). Additional independent t-tests at each time point revealed significantly greater FFA levels at 30 minutes (p = 0.001) and 60 minutes (p < 0.001) in the TD group in comparison to the PLA group (Figure 3). Interestingly, both groups also experienced significantly greater changes in FFA concentrations at all time points following supplement ingestion (p < 0.05). Furthermore, there was a weak but significant correlation between caffeine dosage within the TD group on day 28 and FFA AUC (*r *= 0.40, p = 0.001)

There was no difference in day 28 REE AUC values between groups (p = 0.21; Figure 3). Note that the TD group presented significantly greater changes in REE levels at all time points following supplement ingestion (p ≤ 0.001), whereas the PLA group presented significantly greater values 120 (p = 0.004) and 180 min post-ingestion (p = 0.008). Interestingly, there was no correlation between caffeine dosage within the TD group on day 28 and REE AUC (r = -0.37, p = 0.057).

### Attenuation of thermogenic and lipolytic effects

A significant group × time interaction existed between day 0 and day 28 glycerol AUC values (p = 0.045). Independent t-tests revealed that glycerol AUC values were not different between groups at day 0 (p = 0.18), or day 28 (p = 0.21) (Figure 1). Within group comparisons between days 0 and 28 revealed that glycerol AUC values were significantly higher at day 0 compared to day 28 in the TD group (p = 0.03), whereas there were no changes in the PLA group at day 28 compared to day 0 (p = 0.46). Further, glycerol values were higher on day 0 prior to and 30, 120, and 180 minutes following drink consumption within the TD group when comparing days 0 and 28 (p < 0.05) (Figure 1).

No significant group × time interaction existed between day 0 and day 28 for FFA AUC values (p = 0.70). Independent t-tests revealed that FFA AUC values were greater in the TD group at days 0 (p = 0.012) and 28 (p = 0.048) (Figure 2). Within-group comparisons between days 0 and 28 revealed that FFA AUC values did not change in the TD group (p = 0.60), nor did they change in the PLA group (p = 0.98). A within-group comparison revealed that FFA levels in the TD group were greater at 120 min on day 0 compared to day 28 (p = 0.02) (Figure 2).

Finally, a trend for a group × time interaction existed between day 0 and day 28 REE AUC values (p = 0.053). Independent t-tests revealed that REE AUC values were significantly greater in the TD group versus the PLA group at day 0 (p = 0.05), but not at day 28 (p = 0.28) (Figure 3). Within group comparisons between days 0 and 28 revealed that there was a significant decrease in REE AUC values in the TD group (p = 0.04), although there was no change in the PLA group (p = 0.43) following prolonged supplementation. Further, there were no differences between REE values at any time point within the TD group when comparing days 0 and 28 (Figure 3).

### Clinical safety

Changes in complete blood cell and clinical serum chemistry markers are presented in Table 2. There were no significant group × time interactions for red blood cell count, white blood cell count, or white blood cell differentials over the course of the study (p > 0.05). Furthermore, no significant group × time interactions (p > 0.05) existed for serum triglycerides, HDL cholesterol, LDL cholesterol, glucose, hepatic enzymes (i.e., AST, ALT), hepatic proteins (i.e., total protein, albumin, bilirubin), electrolytes (i.e., sodium, potassium, chloride, calcium, carbon dioxide), or crude markers of kidney integrity (i.e., ALP, BUN, creatinine). All reported main effects over time were within clinically accepted normative data [13]. Likewise, no significant (p > 0.05) interactive or main effects were reported for systolic blood pressure, diastolic blood pressure, or heart rate.

**Table 2:** Changes in clinical serum chemistry and complete blood count variables

Variable	Group	Day 0	Day 28	Significance	
Triglycerides (mg·dl^-1^)	TD PLA	94 ± 8 94 ± 7	105 ± 9 98 ± 7	Group: Time: G × T:	0.77 0.10 0.44
Total Chol (mg·dl^-1^)	TD PLA	162 ± 5 165 ± 6	164 ± 5 168 ± 5	Group: Time: G × T:	0.61 0.32 0.99
HDL Chol (mg·dl^-1^)	TD PLA	58 ± 3 54 ± 2	57 ± 3 54 ± 2	Group: Time: G × T:	0.33 0.92 0.44
LDL Chol (mg·dl^-1^)	TD PLA	85 ± 4 94 ± 6	86 ± 5 94 ± 5	Group: Time: G × T:	0.26 0.73 0.91
Glucose (mg·dl^-1^)	TD PLA	94 ± 1 91 ± 1	96 ± 1 92 ± 1	Group: Time: G × T:	0.07 0.06 0.52
Sodium (mM)^†^	TD PLA	141 ± 1 141 ± 1	142 ± 2 142 ± 2	Group: Time: G × T:	0.93 0.03 0.96
Potassium (mM)	TD PLA	4.4 ± 0.1 4.5 ± 0.1	4.5 ± 0.1 4.6 ± 0.1	Group: Time: G × T:	0.20 0.41 0.96
BUN (mg·dl^-1^)	TD PLA	14 ± 1 14 ± 1	14 ± 1 13 ± 1	Group: Time: G × T:	0.63 0.35 0.36
Creatinine (mg·dl^-1^)^†^	TD PLA	1.0 ± 0.0 1.0 ± 0.0	1.0 ± 0.0 1.0 ± 0.0	Group: Time: G × T:	0.46 0.007 0.42
ALT (U·L^-1^)	TD PLA	20 ± 2 19 ± 1	18 ± 1 18 ± 1	Group: Time: G × T:	0.69 0.12 0.51
AST (U·L^-1^)	TD PLA	20 ± 1 20 ± 1	20 ± 1 21 ± 2	Group: Time: G × T:	0.94 0.53 0.74
RBC Count (10^6^·μl^-1^)^†^	TD PLA	5.00 ± 0.12 4.91 ± 0.14	4.63 ± 0.08 4.62 ± 0.10	Group: Time: G × T:	0.75 < 0.001 0.68
Hemoglobin (g·dl^-1^)^†^	TD PLA	14.5 ± 0.2 14.8 ± 0.3	14.2 ± 0.2 14.4 ± 0.3	Group: Time: G × T:	0.42 0.003 0.50
Hematocrit (%)^†^	TD PLA	43.6 ± 1.6 42.7 ± 0.8	40.8 ± 0.7 41.0 ± 0.9	Group: Time: G × T:	0.78 0.02 0.56
WBC Count (10^3^·μl^-1^)	TD PLA	5.9 ± 0.2 5.9 ± 0.2	5.8 ± 0.2 6.1 ± 0.2	Group: Time: G × T:	0.63 0.95 0.36

TD, thermogenic drink group (n = 30)

PLA, placebo group (n = 30)

Values are expressed as means ± SE

^†^Significant main effect for time, p < 0.05

## Discussion

The aim of this study was to examine how prolonged TD use affected the acute thermogenic and lipolytic actions upon ingestion as well as long-term changes in body composition blood safety parameters. Day 28 REE AUC values were not significantly different in the TD versus the PLA group (p > 0.05) suggesting that changes in REE were similar in both groups at the end of the protocol. Furthermore, while day 28 REE values within the TD group were significantly increased at all time points in comparison to baseline, these values (within TD group only) for REE and glycerol at day 0 were significantly greater than day 28 values. Conversely, FFA AUC levels at day 0 and day 28 in the TD group were significantly greater than the PLA group, and FFA levels did not trend downward over the 28-day supplementation period. Finally, TD ingestion did not alter blood safety markers and was responsible for a significant reduction in body fat levels after 28 days of supplementation.

### Day 28 versus day 0 energy expenditure changes

Acute TD ingestion has been shown to increases REE by 12% when compared a PLA treatment resulting in an average of 120 more calories being expended over a 3-hour time period [7]. However, while REE values in the present study continued to significantly increase REE in the TD group on day 28, AUC values on day 28 for REE within the TD group were significantly lower than day 0 values providing evidence of a habituation effect. Similarly, Dekker et al. [14] demonstrated that consuming 5 mg·kg^-1 ^caffeine increased serum FFA and plasma adrenaline concentrations. In accordance with REE and glycerol data from the present study, these authors also reported a habituation effect (i.e., a diminished appearance of FFA and adrenaline) following 14 days of chronic caffeine consumption at a dosage of 5 mg·kg^-1^. Similarly, Van Soeren et al. [15] demonstrated that habitual caffeine users did not present increases in circulating epinephrine following acute caffeine ingestion. In contrast, previous literature has also demonstrated that a mixture of caffeine and green tea extract increases REE in humans at the start of and after 8 weeks of supplementation [6]. Data from the present study supports previous literature suggestive of a habituation effect in REE levels. The lack of statistical difference concerning REE values between groups should be interpreted with caution as the PLA group experienced significant increases in REE levels at 120 and 180 minutes after drink ingestion on day 28, which were unexpected (Figure 3; sub-figure B). To this point, significant within-group elevations in REE levels were evident within the TD group at 60, 120 and 180 minutes suggesting that TD ingestion stimulated increases in REE. However, when comparing the entire 3-hour response of REE values at day 0 and day 28 using area under the curve analysis, no significant differences (p > 0.05) were found within the TD group. This data leads the authors to conclude that prolonged ingestion of the TD did result in an attenuation of REE changes.

### Day 28 versus day 0 changes in lipolysis markers

The lipolytic mechanisms associated with caffeine ingestion include the following [16]: 1) central nervous system stimulation resulting in an increase in circulating epinephrine, 2) an inhibition in intracellular phosphodiesterase activity leading to sustained intracellular cAMP and activated perilipin and HSL levels, and 3) the antagonism of peripheral adenosine (A_1_) receptors which further increases intracellular cAMP levels [17]. Furthermore, EGCG has been shown to increase thermogenesis in brown adipocytes through beta-adrenergic receptor stimulation [18], as well as increase circulating markers of lipolysis in mice [19]. However, prolonged caffeine consumption may result in the development of a metabolic tolerance whereby the central nervous system and peripheral tissue stimulation is blunted in chronic users [9]. For instance, analysis of rat adipocytes following prolonged *ad libitum *caffeine ingestion (i.e., 14–16 days) revealed that caffeine up-regulates adenosine (A_1_) receptor content by 37% [9]; this effect being detrimental for HSL activation. Moreover, Robertson *et al. *[8] reported that although administering 250 mg caffeine acutely increased plasma epinephrine levels in humans, prolonged caffeine administration (i.e., 250 mg·day^-1 ^for 7 days) blunted these increases during a follow-up administration. The present study did not examine the humoral and/or intra-adipocyte events that occurred with prolonged TD use. Nonetheless, FFA AUC did not decrease at day 28 when compared to day 0 within the TD group (p = 0.60) to suggest that FFA kinetics were not attenuated as a result of prolonged TD ingestion. AUC data for glycerol, however, was found to be attenuated as day 28 AUC values were significantly lower than day 0 AUC values (p = 0.03). These data provide conflicting evidence regarding lipolysis activity after prolonged TD ingestion and provide a partial explanation for the observed changes in body composition. While FFA liberation appears to be sustained following 28 days of TD supplementation, it is possible that not all of the liberated FFAs were oxidized. Acheson et al. [20] used an isotopic palmitate infusion method and found that acute caffeine ingestion (10 mg per kg body weight) increased energy expenditure 13% over a 3-hour period (similar to the present study) and doubled the liberation of serum lipids in healthy young males. However, these authors reported that 24% of liberated lipids were oxidized whereas 76% were systemically re-esterified suggesting that this phenomenon may have limited the actual oxidation of adipose tissue in the present study. Data from the present study is limited, however, in that it only reflects adaptations over a 28 day period. Whether a complete attenuation effect develops following longer ingestion periods remains to be determined.

### Changes in body composition

While TD supplementation tended to change %BF levels and decrease fat mass over the 28-day intervention when compared to the PLA group, caution must be exercised when interpreting these results. First, *post hoc *analysis revealed a non-significant increase in %BF (p = 0.13) within the PLA group which may have contributed to the significant differences between groups. Whether this increase was the result of changes in physical activity is unknown as no changes in dietary intake were found in this group. Nonetheless, a trend for %BF levels to decrease (p = 0.09) and a significant decrease in fat mass (p = 0.01) within the TD group was also observed. While past literature has demonstrated that caffeine/EGCG mixtures are successful at sustaining weight loss [6,21], these studies employed supplementation periods that were longer in duration (i.e., 8–12 weeks). Specifically, Belza *et al. *[6] demonstrated that a combination of caffeine, catechins, capsaicin, and tyrosine further reduced body fat content by 0.9 kg over an 8-week period after an initial 4-week weight loss period as determined by dual x-ray energy absorptiometry in overweight/obese individuals. Participants in the TD group lost 0.6 kg of fat mass in the present study which is similar to these previously published studies. Using data from the present study, the likely explanation for the changes in body composition relate to the increase in REE and FFA, however, the magnitude of fat mass changes suggests additional thermogenic influences. In this regard, it is possible that TD ingestion spontaneously increased the physical activity levels as this has been found to occur in rodents via the effect that caffeine has on blunting CNS adenosine receptors [22]. The authors are confident that the reported change in body composition was not the result of poor internal stability from the body composition measurement. In this regard, the minimum difference between body composition changes (i.e., the %BF differences between the TD and PLA groups after 28 day) should exceed the standard error of measurement (SEM) for the measuring device in order to be considered a real difference [23]. In this regard, we detected a 1.18 %BF difference between groups whereas our lab has shown the SEM for the BOD POD to be 0.48 %BF (ICC *r *> 0.99, p < 0.001) (*unpublished observations*). Consequently, TD ingestion for 28 days resulted in significantly lower levels of fat mass and a tendency for %BF levels to improve when compared to PLA. Regardless, it is the authors contention that longer investigations in conjunction with dietary and exercise regimens need to be conducted to more accurately ascertain the role a TD may have at improving body composition and improving fitness parameters.

## Conclusion

Acute changes in REE and markers of lipolysis (e.g. free-fatty acids and glycerol) were mixed in their response to acute ingestion at baseline and after 28 days of ingestion. FFA levels were significantly increased from baseline at day 0 and at day 28 suggesting that prolonged ingestion of a TD does not attenuate this response. An attenuation effect was evident, however, in the REE and glycerol data as both variables did not result in significant changes after 28 days of ingestion. While largely preliminary, TD ingestion may help to stimulate fat loss as those individuals who consumed the TD experienced a significant reduction in fat mass and an improvement in %BF. These findings should be carefully considered as the present study only spanned 28 days and did not invoke dietary or exercise intervention. Further, daily TD ingestion was not responsible for any significantly alterations in hemodynamic parameters and/or serum clinical chemistry/CBC markers. Future studies should also examine the acute and prolonged effects of the TD versus similar dosages of the active ingredients (e.g. caffeine, EGCG, etc.) to determine their individual and synergistic impact. Moreover, follow-up studies concerning the effects that TD supplementation has on changes in body composition and/or fitness parameters (i.e., strength, maximal oxygen uptake, rate of perceived exertion, time-to-exhaustion, etc.) in concert with a diet/exercise regimen should be examined in sedentary, overweight/obese individuals.

## Abbreviations

%BF: body fat percentage; A_1_: type I adenosine receptor; AUC: area under the curve; C_*v*_: coefficient of variation; cAMP: 3'-5'-cyclic adenosine monophosphate; EGCG: epigallocatechin; FFA: free fatty acid; OTC: over-the-counter; PLA: non-caloric placebo group; SEM: standard error of measurement; TD: thermogenic energy drink group.

## Competing interests

Celsius Inc. (Delray Beach, FL) provided funding for this project through an unrestricted research grant to the University of Oklahoma. All researchers involved independently collected, analyzed, and interpreted the results from this study and have no financial interests concerning the outcome of this investigation. Publication of these findings should not be viewed as endorsement by the investigator, the University of Oklahoma or the editorial board of the Journal of International Society of Sports Nutrition.

## Authors' contributions

MR was the primary author, oversaw all aspects of study including recruitment, data/specimen analysis, and manuscript preparation. VD was the co-author, oversaw all aspects of study including recruitment, data/specimen analysis, and manuscript preparation. SH was the co-author, assisted with data collection, data analysis and manuscript preparation. JS was the study co-investigator who helped design study and assisted with data collection, statistical analyses, and manuscript preparation. CK was the principal investigator of the study who obtained grant funds for project, designed study, supervised data collection and analysis, supervised statistical analyses, and assisted with manuscript preparation.
